# *Candida haemulonii* species complex: an emerging species in India and its genetic diversity assessed with multilocus sequence and amplified fragment-length polymorphism analyses

**DOI:** 10.1038/emi.2016.49

**Published:** 2016-05-25

**Authors:** Anil Kumar, Anupam Prakash, Ashutosh Singh, Harish Kumar, Ferry Hagen, Jacques F Meis, Anuradha Chowdhary

**Affiliations:** 1 Department of Microbiology, Amrita Institute of Medical Sciences, Ponekara, Kochi, Kerala 682041, India; 2 Department of Medical Mycology, Vallabhbhai Patel Chest Institute, University of Delhi, Delhi 110007, India; 3 Department of Endocrinology, Diabetes and Podiatric Surgery, Amrita Institute of Medical Sciences, Ponekara, Kochi, Kerala 682041, India; 4 Department of Medical Microbiology and Infectious Diseases, Canisius-Wilhelmina Hospital, Nijmegen 6532SZ, The Netherlands; 5 Department of Medical Microbiology, Radboudumc, Nijmegen 6525GA, The Netherlands

**Dear Editor,**

The epidemiology of *Candida* species-associated invasive fungal infections is evolving, and many uncommon *Candida* species have recently emerged as etiologic agents of bloodstream and other invasive infections. This emergence is attributable to the use of antifungal drugs, such as azoles, for prophylaxis and echinocandins among high-risk populations. Notably, these species exhibit decreased *in vitro* susceptibility to the antifungals used for therapy.^1^ In the last few years, clinical treatment failures for *Candida haemulonii* infections associated with resistance to amphotericin B (AMB) and reduced susceptibility to azoles and echinocandins have been reported.^2, 3, 4^ Members of the *Candida haemulonii* species complex are uncommon yeasts that cause bloodstream and deep-seated infections, and consist of two genotypically distinguishable species, that is, *C. haemulonii* and *C. duobushaemulonii*, and a variety, *C. haemulonii* var. *vulnera.*^2^ These species and other relatives of *C. haemulonii,* that is, *Candida auris* and *Candida pseudohaemulonii*, cannot be differentiated by the commercial yeast identification methods used in microbiology laboratories; thus, the true distributions of *C. haemulonii* and sibling species remain unknown.^5^

Although the prevalence of uncommon *Candida* species vary geographically, infections due to *C. haemulonii* are primarily reported in South America, Asia, the Middle East and Europe.^2, 3, 4, 5, 6, 7, 8^ Despite their emergence as opportunistic yeasts in several countries, there is a paucity of data regarding the genetically related *C. haemulonii* species complex from clinical sources. In addition, unlike its sibling species, *C. auris*, which is known for clonal endemicity in hospitals, no information regarding the genetic diversity of the *C. haemulonii* species complex is available.^5^ Herein, we describe the emergence of the *C. haemulonii* species complex in three hospitals in India. Furthermore, multilocus phylogenetic analyses were performed to investigate the species/varieties in the clinical *C. haemulonii* species complex, and amplified fragment-length polymorphism (AFLP) fingerprinting was applied to determine their genetic diversity. AFLP was first described 20 years ago and is known to have high discriminatory power due to the generation of many informative genetic markers.^9^ In addition, the antifungal susceptibility profile of the *C. haemulonii* species complex was determined.

Clinical isolates were collected from 2010 to 2015 from individual patients in two tertiary care hospitals in Delhi and a solitary hospital in Kochi, Kerala, in South India. The isolates were presumptively identified as *C. haemulonii* with the VITEK2 system (Biomerieux, Marcy l'Etoile, France) in local hospitals. However, matrix-assisted laser desorption ionization–time-of-flight mass spectrometry (MALDI–TOF MS; Bruker-Daltonics, Bremen, Germany) accurately identified the species and variety in the *C. haemulonii* species complex. These species included *C. duobushaemulonii* (*n*=8), *C. haemulonii* (*n*=6) and *C. haemulonii* var. *vulnera* (*n*=1). The species' morphologies were studied on Sabouraud dextrose agar, CHROMagar Candida medium (Becton-Dickinson, Baltimore, MI, USA) and rice Tween-80 agar, and their growth characteristics were examined at 37 °C and 42 °C. Antifungal susceptibility testing with fluconazole (FLU), itraconazole (ITC), voriconazole (VRC), isavuconazole (ISAV), posaconazole (POS), AMB, 5-flucytosine (FC), caspofungin (CAS), micafungin (MFG) and anidulafungin (AFG) was performed using both the M27-A3 Clinical and Laboratory Standards Institute (CLSI) broth microdilution method^10^ and the AST-YS07 card of the VITEK2. Furthermore, to determine the multilocus phylogeny, four loci, including the internal transcribed spacer region (ITS), D1/D2, *RPB1* and *RPB2,* were sequenced.^2^ A neighbor-joining (NJ) phylogenetic tree based on the concatenated sequences of the four loci was constructed using MEGA version 6 (http://www.megasoftware.net/). In addition, as previously described, an AFLP dendrogram was generated with Bionumerics v6.6 (Applied-Maths, Sint-Martens-Latem, Belgium) using the standard Pearson and unweighted pair group method with averages (UPGMA).^11^

GenBank BLAST searches of the ITS region and MALDI–TOF MS confirmed the presence of 15 isolates belonging to *C. duobushaemulonii* (*n*=8), *C. haemulonii* (*n*=6) and *C. haemulonii* var. *vulnera* (*n*=1). The ITS sequences of all of the isolates exhibited 100% homologies with the type and reference strains of the respective species in GenBank. The isolates originated from the deep-seated tissue or bone obtained during surgical debridement (*n*=9), blood (*n*=5) and BAL (*n*=1). The isolates exhibited budding yeast cells with pseudohyphae and developed a dark pink color in CHROMagar Candida medium after 48 h of incubation at 37 °C. The isolates grew well at 37 °C, whereas no growth was observed at 42 °C. The NJ tree consisted of three major clades (Figure 1). Clades 1 and 2 contained *C. haemulonii* (*n*=3 each) and a solitary *C. haemulonii* var. *vulnera* isolate in addition to the type and reference strains, and suggested strain variation. The similarity percentages among the *C. haemulonii* isolates were 79%–89%. However, all of the *C. duobushaemulonii* clustered together in clade 3. Notably, and as previously reported, the currently available loci (ITS, LSU, *RPB1* and *RPB2*) possessed low discriminatory power for describing the intra-species genetic diversity of *C. haemulonii*, *C. haemulonii* var. *vulnera* and *C. duobushaemulonii*.^2^ Thus, the inclusion of better polymorphic genes for multilocus sequence analysis is warranted for investigations of the clonal transmissions of these species. The AFLP fingerprint analysis revealed markedly variable banding patterns with ~50 bands/strain comprising 25–425 bp overall.

High geometric mean (GM) minimum inhibitory concentrations (MICs) for FLU (CLSI 12.7 mg/L; VITEK2 24.2 mg/L) and AMB (CLSI 14.6 mg/L; VITEK2 6.6 mg/L) were observed via both the CLSI and VITEK2 methods. In contrast, all isolates exhibited low CLSI GM MICs for ISA (0.02 mg/L), POS (0.08 mg/L), ITC (0.3 mg/L) and VRC (0.134 mg/L) with the exception of a solitary *C. haemulonii* isolate that exhibited a high MIC for VRC (4 mg/L). Notably, a significant twofold greater CLSI MIC for FLU was observed for *C. haemulonii* (GM 26.2 mg/L) than for *C. duobushaemulonii* (GM 6.72 mg/L; Supplementary Table S1). In addition, all of the isolates exhibited low GM MICs for CAS (CLSI 0.15 mg/L; VITEK2 0.30 mg/L), MFG (CLSI 0.33 mg/L; VITEK2 0.10 mg/L) and AFG (CLSI 0.5 mg/L), with the exception being solitary isolates of *C. haemulonii* var. *vulnera* and *C. duobushaemulonii,* which exhibited high MICs (1 mg/L) against all of the echinocandins and high MICs (1 mg/L) for AFG and MFG, respectively, by CLSI. Furthermore, significantly lower (Mann-Whitney *P*<0.05) GM MICs for VRC, CAS and FC, and higher GM MICs for AMB and MFG were observed by CLSI compared with VITEK2. However, the two methods exhibited good agreement (80%–100%) for AMB, FLU, CAS and MFG, but a low agreement (20%) was noted for VRC. Therefore, standardization of VITEK2 via testing with a larger number of *C. haemulonii* isolates is warranted to validate the use of the VITEK2 system as an alternative method for routine use in laboratories for susceptibility testing.

The clinical features and outcomes of the 15 patients were retrieved from records (Supplementary Table S2). Nine patients had chronic foot infections, and surgically debrided bone and soft tissues yielded *C. haemulonii* isolates that included *C. haemulonii* var. *vulnera* in five patients and *C. duobushaemulonii* in four. All nine patients had the major risk factor of uncontrolled diabetes and underwent foot amputations. Two of the nine patients also received azole antifungals. Among the five candidemia patients, two had acute myeloid leukemia and the remaining three patients had the underlying conditions of renal transplant and abdominal surgery. Breakthrough candidemia while on azole prophylaxis (FLU=3 and VRC=1) was noted in four patients. VRC therapy was initiated in three candidemia patients and two were successfully managed. The 28-day all-cause mortality among the candidemia patients was 60%. Overall, the patients had at least three predisposing risk factors for candidiasis as previously reported,^12^ and these risk factors included the following: 15 (100%) were on broad-spectrum antibiotics, ten (66%) had diabetes mellitus, six (40%) had chronic kidney conditions, five (33.3%) had coronary artery disease, four (27%) had peripheral occlusive vascular disease, three (20%) had malignancies, two (13%) had undergone renal transplant and one had undergone abdominal surgery.

Worldwide, the uncommon *Candida* species that cause infections are not well characterized.^1, 13^ The present study reports the large series of candidiasis cases due to the *C. haemulonii* species complex with clinical and microbiological data that provide novel insights into this yeast. Unlike its sibling species, *C. auris*, which is widely prevalent in Indian hospitals, *C. haemulonii* accounts for only 0.8% of *Candida* species. Notably, previous reports regarding the occurrence of *C. haemulonii* in India are misleading because the identifications were performed with conventional or commercial identification systems.^14^ Recently, Kathuria *et al.*^15^ reported that of 102 *C. haemulonii* isolates identified with the VITEK2 system in five centers in India, 88.2% (*n*=90) were subsequently confirmed as *C. auris* by ITS sequencing.

Notably, a high frequency of infections due to the *C. haemulonii* species complex was observed in the patients with diabetes mellitus (66.6%). Furthermore, as previously reported, the *C. haemulonii* isolates exhibited elevated MICs to AMB and FLU.^2, 3^ In addition, the high MICs to echinocandins noted in two isolates (*C. haemulonii* var. *vulnera* and *C. duobushaemulonii*) are worrisome. Thus far, two previous studies have reported high MICs for echinocandins.^2, 4^ In conclusion, this study highlights the significance of *C. haemulonii* species in chronic foot infections and candidemia, and the cases of the latter were associated with breakthrough infections, specifically in the patients on FLU therapy. Thus, correct identifications and determinations of the antifungal susceptibilities of *C. haemulonii* isolates are warranted to guide antifungal therapy. Because the clinical experience with *C. haemulonii* infections is limited, optimal treatments have not yet been defined.^16^ However, the *in vitro* activities of VRC, POS and ISAV suggest that they can be used as effective treatment options.

## GenBank nucleotide sequence accession numbers

ITS, KP862806-KP862817 and KU361136-KU361138; D1/D2, KU361120-KU361134; *RPB1,* KU361809-KU361103; and *RBP2,* KU3361104-KU361118.

## Figures and Tables

**Figure 1:**
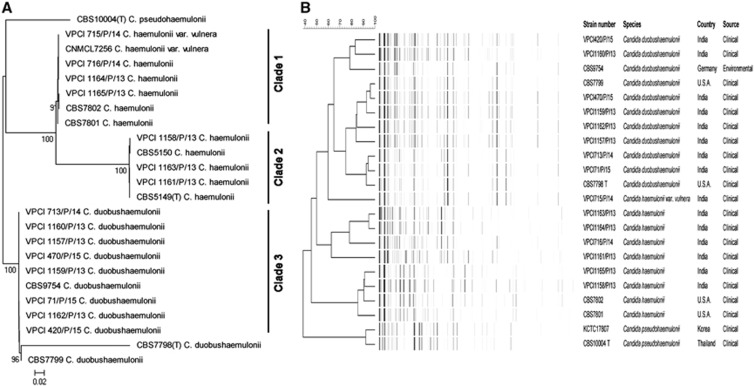
(**A**) Neighbor-joining phylogenetic tree based on the ITS, D1/D2, *RPB1* and *RPB2* sequences with 2000 bootstrap replications using MEGA version 6. (**B**) AFLP dendrogram using UPGMA in combination with the Pearson correlation coefficients of the *Candida haemulonii* species complex and the reference strains (*C. duobushaemulonii* CBS9754, CBS7799 and CBS7798^T^; *C. haemulonii* CBS7801 and CBS7802; and *C. pseudohaemulonii* CBS 10004^T^ and KCTC17807). The scale bar indicates the percentage similarity. amplified fragment-length polymorphism, AFLP; internal transcribed spacer region, ITS; unweighted pair group method with averages, UPGMA.
