# Supplementary material for: Candida haemulonii species complex: an emerging species in India and its genetic diversity assessed with multilocus sequence and amplified fragment-length polymorphism analyses
Source: Emerg Microbes Infect. 2016 May 25;5(5):e49–. doi: 10.1038/emi.2016.49 (PMC4893546; doi:10.1038/emi.2016.49)
Supplement: Supplementary Table S1 [file emi201649x1.pdf]

Supplementary Table S1 ***In-vitro*** **antifungal susceptibility profile of** ***Candida haemulonii*** **complex isolates (*n*=15) against azoles, echinocandins and amphotericin B by using CLSI and VITEK 2[*]**

Species or MIC parameter (mg/L)	Drugs[a]									
	**AMB**	**FLU**	**ITC**	**VRC**	**ISAV**	**POS**	**FC**	**CAS**	**MFG**	**AFG**
***C. duobushaemulonii* (*n*=8)**										
MIC_{50} [b]	16 (8)	8 (16)	0.25	0.125 (2.5)	0.03	0.125	0.125 (1)	0.125 (0.25)	0.5 (0.125)	0.5
MIC_{90} [c]	16 (16)	16 (64)	0.5	0.125 (8)	0.06	0.125	0.125 (1)	0.25 (0.5)	0.5 (0.125)	0.5
GM[d]	16 (9.5)	6.72 (26.9)	0.27	0.08 (2.5)	0.03	0.10	0.125 (1)	0.13 (0.27)	0.38 (0.09)	0.54
Range	16 (4-16)	1-16 (16-64)	0.25-0.5	0.03-0.125 (1-8)	0.015-0.06	0.03-0.125	0.125 (1)	0.06-0.25 (0.25-0.5)	0.125-1 (0.06-0.125)	0.5-1
***C. haemulonii,* (*n*=6), *C. h.* var. *vulnera* (*n*=1)**										
MIC_{50}	16 (8)	64 (16)	0.25	0.5 (0.125)	0.015	0.125	0.125 (1)	0.125 (0.25)	0.25 (0.125)	0.5
MIC_{90}	16 (8)	64 (64)	0.5	2 (4)	0.25	0.25	64 (64)	1 (0.5)	0.5 (0.5)	0.5
GM	13.12 (4)	26.2 (21.5)	0.33	0.25 (0.54)	0.03	0.06	1.8 (5.3)	0.18 (0.33)	0.27 (0.11)	0.45
Range	4-16 (1-8)	2-64 (4-64)	0.25-0.5	0.03-4 (0.125-4)	0.015-0.5	0.015-0.25	0.125-64 (1-64)	0.06-1 (0.25-0.5)	0.125-1 (0.06-0.5)	0.25-1
**Total**										
MIC_{50}	16 (8)	8 (16)	0.25	0.125 (1)	0.015	0.125	0.125 (1)	0.125 (0.25)	0.25 (0.125)	0.5
MIC_{90}	16 (16)	64 (64)	0.5	0.5 (8)	0.06	0.25	64 (64)	0.25 (0.5)	0.5 (0.125)	0.5
GM	14.8 (6.5)	16 (24.25)	0.314	0.134 (1.25)	0.03	0.08	0.435 (2.19)	0.156 (0.3)	0.33 (0.1)	0.5
Range	4-16 (1-16)	1-64 (4-64)	0.25-0.5	0.03-4 (0.125-8)	0.015-0.5	0.015-0.25	0.125-64 (1-64)	0.06-1 (0.25-0.5)	0.125-1 (0.06-0.5)	0.25-1

[*]figures in parenthesis indicate MICs data obtained from VITEK2.[a]AMB, Amphotericin B; FLU, fluconazole; ITC, itraconazole; VRC, voriconazole; ISAV, isavuconazole; POS, posaconazole; FC, 5-flucytosine; CAS, caspofungin; MFG, micafungin; AFG, anidulafungin; [b]MIC_{50} at which 50% of test isolates inhibited; [c]MIC_{90} at which 90% of test isolates inhibited; [d]Geometric mean of MICs;
