# Supplementary material for: Candida haemulonii species complex: an emerging species in India and its genetic diversity assessed with multilocus sequence and amplified fragment-length polymorphism analyses
Source: Emerg Microbes Infect. 2016 May 25;5(5):e49–. doi: 10.1038/emi.2016.49 (PMC4893546; doi:10.1038/emi.2016.49)
Supplement: Supplementary Table S2 [file emi201649x2.pdf]

Supplementary Table S2 Clinical evaluation of 15 cases due to *Candida haemulonii* species complex.

**Characteristics**	***Candida duobushaemulonii* (*n*=8)**	***Candida haemulonii* (*n*=6)and *C. h.* var. *vulnera* (*n*=1)**
Age mean years (range)	53 (23-79)	59.7 (10-79)
Sex (male/female)	6/2	6/1
Days of hospitalization mean (range)	24.3 (9-50)	20.14 (8-50)
**Baseline diseases**		
Diabetes mellitus	5 (63%)	5 (71%)
Hematologic malignancy	2 (25%)	1 (14%)
Vascular disease	1 (13%)	3 (43%)
Chronic kidney disease	2 (25%)	4 (57%)
Chronic artery disease	1 (13%)	4 (57%)
Solid organ transplant	1 (13%)	1 (14%)
Hypertension	4 (50%)	4 (57%)
Acute respiratory disease syndrome	1 (13%)	-
Pneumonia	-	1 (14%)
Abdominal surgery	1 (13%)	-
**Risk factor**		
Antifungals (azoles)	2 (FLU[a]), 1(VRC[b])	2 (FLU), 1 (VRC)
Broad spectrum antibiotics	8	7
**Lower extremity wounds**	4 (50%)	5 (71%)
**Candidemia**	3 (38%)	2 (29%)
**Therapy**	1 (FLU), 1 (VRC)	2 (VRC)
**Outcome (cured/death)**	6/2	6/1

[a]Fluconazole, [b] Voriconazole
